# Corneal Perforation associated with “Silent Brain Syndrome”

**DOI:** 10.5935/0004-2749.20230004

**Published:** 2023

**Authors:** Otavio de Azevedo Magalhaes, Mauricio C. Lima, Marcelo Blochtein Golbert, Luiza Birck Klein, Ricardo Mörschbächer

**Affiliations:** 1 Setor de Córnea, Hospital Banco de Olhos de Porto Alegre, Porto Alegre, RS, Brazil; 2 Setor de Oculoplástica, Hospital Banco de Olhos de Porto Alegre, Porto Alegre, RS, Brazil

**Keywords:** Enophthalmos, Corneal perforation, Hydrocephalus, Ventriculoperitoneal shunt, Keratoplasty, penetrating, Orbit/ transplantation, Humans, Case report, Enoftalmia, Perfuração da córnea, Hidrocefalia, Derivação ventriculoperitoneal, Ceratoplastia penetrante, Órbita/ transplante, Humanos, Relato de caso

## Abstract

This case report describes the clinical characteristics and ophthalmic management of a patient who developed corneal perforation due to severe enophthalmos consistent with “silent brain syndrome.” A 27-year-old man with a history of congenital hydrocephalus and ventriculoperitoneal shunt was referred with complaints of “sinking of the eyeballs” and progressively decreasing vision in the left eye. Examination revealed severe bilateral enophthalmos in addition to superonasal corneal perforation with iris prolapse in the left eye. The patient underwent therapeutic keratoplasty the next day. Orbital reconstruction with costochondral graft and shunt revision of the intracranial hypotension were performed the next month to prevent further progression.

## Abstract (pt)

Este relato de caso descreve as características clínicas e o manejo cirúrgico de um paciente que teve perfuração da córnea devido à enoftalmia grave consistente com a “síndrome do cérebro silencioso”. Um homem de 27 anos com história de hidrocefalia congênita e derivação ventrículo-peritoneal foi encaminhado com queixas de “afundamento dos globos oculares” e diminuição progressiva da visão no olho esquerdo. O exame revelou enoftalmo bilateral importante, além de perfuração superonasal da córnea com prolapso iriano no olho esquerdo. A paciente foi submetida à ceratoplastia terapêutica no dia seguinte. Foi realizado no mês seguinte a reconstrução da órbita com enxerto costocondral e revisão do shunt para evitar progressão e piora do caso.

## INTRODUCTION

The association between ventriculoperitoneal shunt (VPS) and progressive bilateral enophthalmos was reported for the first time by Meyer et al.^(1)^. A decade later, Cruz et al. proposed that enophthalmos was a specific form of shunt-related skull change secondary to reduction of cerebrospinal fluid pressure that can lead to progressive orbital bone changes^(2)^. Other reports have confirmed these findings^(3,4,5,6,7)^. Even though exposure keratopathy has been described in some of these cases, no corneal perforation has been reported so far. In compliance with the Health Insurance Portability and Accountability Act (HIPPA) guidelines and adherence to the tenets of the Declaration of Helsinki, we now describe a case of corneal perforation associated with severe bilateral enophthalmos after VPS.

## CASE REPORT

**Figure 1.**
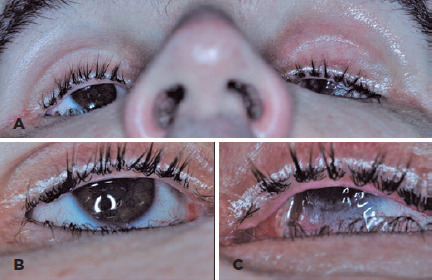
(A) External photographs with close-up view of the medial lower eyelids show loss of apposition of the upper eyelid to the globe due to severe enophthalmos, resulting in OD exposure keratopathy (B) and associated OS conjunctival injection (C).

**Figure 2.**
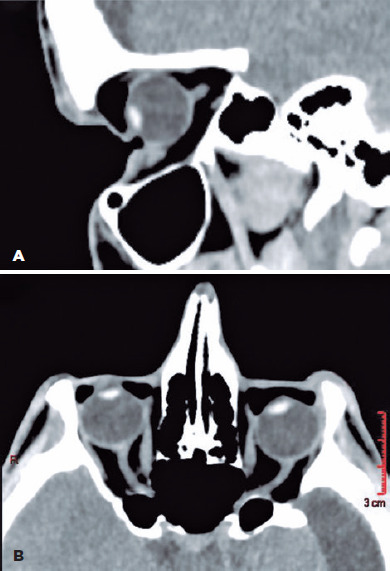
(A) Sagittal and (B) axial CT scans show localization of various abnormal air spaces and deepened upper and lower eyelid sulci pulled into the expanded orbits. The images are also oriented to show the entire orbital and part of the intracanalicular segment of the optic nerves to show the lack of redundant optic nerve length.

**Figure 3.**
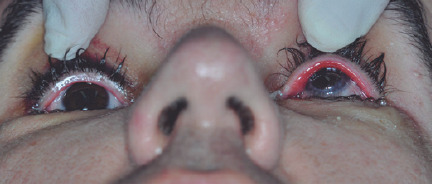
External photograph after orbital augmentation showing reduction of OS enophthalmos.

A 27-year-old Caucasian man with a history of idiopathic congenital hydrocephalus who underwent VPS was referred to the Porto Alegre Eye Bank Hospital with complaints of “sinking of the eyeballs” and ocular irritation in both eyes and a progressive decrease in vision in the left eye (OS). Examination revealed severe bilateral asymmetrical enophthalmos, flat anterior chamber, upper-half erosive keratopathy, and 3-mm superonasal corneal perforation with iris prolapse in the OS. No corneal infiltration or infectious keratitis was observed. Initial examination revealed 20/25 best corrected visual acuity in the right eye (OD) and hand movement in the OS. Ocular motility presented a marked reduction in infraduction OS (Hertel exophthalmometer value of 6 mm OD and 4 mm OS) and eyelid imbrication associated with lower eyelid entropion (Figure 1). The lacrimal drainage system was normal. Severe bilateral enophthalmos due to upward bowing of the orbital roof with significant widespread air under the eyelids, short and straight optic nerves, and enlarged maxillary, ethmoid, and sphenoid sinuses were evident on orbital computed tomography (CT) scan (Figure 2). Under general anesthesia, the patient underwent therapeutic full-thickness 8.25-mm keratoplasty the next morning. The surgical procedure was technically difficult because of the remarkable enophthalmos and restriction of infraduction that created poor exposure. Host graft microbiological cultures were negative for fungal and bacterial infections. Over the next 3 months, penetrating keratoplasty maintained the ocular integrity and improved vision of hand movement to 20/30. The following month, the patient was admitted for shunt revision of intracranial hypotension and for left orbital roof reconstruction with a costochondral graft under general anesthesia through orotracheal intubation. The rib graft was harvested from the right seventh and eighth costal cartilage and contoured in the cavities to correct the shape of the left orbit. The segments were stabilized and accommodated superiorly, inferiorly, nasally, and temporally. An excellent reduction of enophthalmos to a Hertel exophthalmometer value of 10 mm was noted, which restored the contact between the palpebral conjunctiva and left globe, reducing the signs of exposure keratopathy (Figure 3).

## DISCUSSION

Thirteen other reported cases of severe enophthalmos after VPS derived from English ophthalmic literature have been reported (Table 1). All patients were shunted during childhood after hydrocephalus. Moreover, all became symptomatic a few years after shunting and presented symptoms similar to our case. To our knowledge, corneal perforation due to loss of apposition between the upper tarsi and the ocular surface in the presence of complete eyelid closure from progressive marked bilateral enophthalmos has never been described. Further, this is the first description of rib cartilage graft orbital augmentation in the “silent brain syndrome.”

**Table 1.** Age demographics of patients developing bilateral enophthalmos after ventriculoperitoneal shunting (VPS) for childhood hydrocephalus in literature

Patient	First VPS placement*	Most recent VPS replacement or revision*	Ophthalmic presentation*	Reference
1	8	28	33	1
2	0.4	21	24	1
3	14	NA	20	1
4	13	NA	22	2
5	12	NA	24	3
6	14	NA	25	3
7	22	22	25	4,5
8	18	18	19	4,5
9	23	NA	38	4,5
10	11	NA	16	4,5
11	22	24	26	5
12	1	14	33	6
13	3	NA	21	7
Mean	12.4	21.1	25.1	
Range	0.4-23	14-28	16-38	

*Age in years; NA data not available.

It is important to mention that although entropion is associated with enophthalmos, no abrading of the corneal surface from the lower eyelid lashes was observed. This condition was not responsible for the corneal perforation in this case.

Cranial bone growth in childhood is dependent on cerebrospinal fluid pressure, and its reduction with VPS can lead to various skull anomalies^(7)^. Post-shunting enophthalmos was associated with the upward expansion of the orbital roof and sphenoid sinus rather than loss or fibrosis of orbital fat^(6)^. Because of increasing head size, our patient underwent VPS placement at the age of 6 months. He subsequently required four additional revisions, the most recent being at 21 years of age. At that time, sudden neurological deficits including an aphasia and ataxia appeared. CT findings suggested that the patient had an underlying condition of “silent brain syndrome”^(3)^. Shunt removal and correction of the intracranial hypotension may presumably prevent further enophthalmos progression. Implant augmentation of the orbital volume via an upper eyelid crease approach are recommended to restore globe-eyelid apposition^(7)^.

Several cases that have been reported to date, including ours, went undiagnosed for a long time despite frequent ophthalmic and neurosurgical follow-up examinations. This study highlights the importance of VPS monitoring in all patients through a collaborative effort between the ophthalmology and neurosurgery departments.
